# The Role of Non-Coding RNAs in Myelodysplastic Neoplasms

**DOI:** 10.3390/cancers15194810

**Published:** 2023-09-30

**Authors:** Vasileios Georgoulis, Epameinondas Koumpis, Eleftheria Hatzimichael

**Affiliations:** 1 Department of Haematology, University Hospital of Ioannina, Faculty of Medicine, University of Ioannina, 45 500 Ioannina, Greece; vasileios.georgoulis@gmail.com (V.G.); an.koumpis@uoi.gr (E.K.); 2 Computational Medicine Center, Sidney Kimmel Medical College, Thomas Jefferson University, Philadelphia, PA 19 107, USA

**Keywords:** myelodysplastic syndromes, non-coding RNA, microRNA, lncRNA, circRNA, piRNA, tRNA, snoRNA

## Abstract

**Simple Summary:**

Myelodysplastic neoplasms (MDS) are a group of hematologic malignancies with an increased risk of transformation to acute myeloid leukemia. Non-coding RNAs are RNA molecules of variable size that do not translate into proteins but regulate gene expression during multiple cellular processes. These RNAs have been found deregulated in several cancers, including MDS. In this review, we aim to summarize research findings on the biological role of different non-coding RNAs in MDS development and progression, with emphasis on molecules that have exhibited prognostic or predictive value and could, hence, guide decision-making in clinical practice.

**Abstract:**

Myelodysplastic syndromes or neoplasms (MDS) are a heterogeneous group of myeloid clonal disorders characterized by peripheral blood cytopenias, blood and marrow cell dysplasia, and increased risk of evolution to acute myeloid leukemia (AML). Non-coding RNAs, especially microRNAs and long non-coding RNAs, serve as regulators of normal and malignant hematopoiesis and have been implicated in carcinogenesis. This review presents a comprehensive summary of the biology and role of non-coding RNAs, including the less studied circRNA, siRNA, piRNA, and snoRNA as potential prognostic and/or predictive biomarkers or therapeutic targets in MDS.

## 1. Introduction

Myelodysplastic neoplasms (MDS) are a group of myeloid neoplasms characterized by clonal proliferation of hematopoietic stem cells (HSCs) and genetic and epigenetic abnormalities leading to ineffective hematopoiesis, peripheral cytopenias, and a propensity to the development of acute myeloid leukemia (AML) [1,2]. Diagnosis is based on full blood count parameters, bone marrow morphology and blast count, and the presence of cytogenetic and molecular abnormalities, mainly mutations [2]. The most recent World Health Organization (WHO) classification, the fifth edition, recognizes two main groups: a. MDS with defining genetic abnormalities and b. MDS, morphologically defined [3]. Following correct diagnosis and accurate classification, prognosis estimation and risk stratification are crucial to tailor therapy. The revised International Prognostic Scoring System (IPSS-R) is widely used for the risk stratification of MDS patients considering the number and depth of cytopenias and cytogenetic abnormalities [4]; while most recently the molecular IPSS (IPSS-M) combined genomic aberrations with hematologic and cytogenetic abnormalities and provided improved risk stratification of patients with MDS [5]. In general, low-risk patients are managed either expectantly or with recombinant human erythropoietin or luspatercept [6], whereas high-risk patients are offered hypomethylating agents (HMAs) and/or allogeneic hematopoietic stem transplantation (AlloSCT), which remains the only curative modality. Despite all this progress, there is currently no widely accepted predictive model nor a serviceable biomarker of response that can offer a timely and valid estimation of the expected benefit from these available treatment options.

In terms of pathophysiology, genes regulating epigenetic modifications seem to be the most commonly mutated in patients with MDS [7]. Among epigenetic modifiers, non-coding RNA molecules, especially microRNAs (miRNAs) and long non-coding RNAs (lncRNAs) have recently attracted research interest. Until recently, it was believed that the molecules important for the functioning of a cell are those described by the “Central Dogma” of biology, namely messenger RNAs and proteins. However, almost three decades ago, the discovery of microRNAs (miRNAs) in plants [8] and animals [9,10] changed this perception. Subsequent research efforts have demonstrated that large parts of an organism’s genome are transcribed into RNA at one time point or another but are not translated into an amino acid sequence. These RNA transcripts have been referred to as non-coding RNAs (ncRNAs). There are many recognizable classes of ncRNA, each having a distinct function. These include the abovementioned miRNAs, transfer RNAs (tRNAs) [11], ribosomal RNAs (rRNAs) [12], PIWI-interacting RNAs (piRNAs) [13], small nucleolar RNAs (snoRNAs) [14], long intergenic ncRNAs (lincRNAs) [15], etc. The full extent of distinct classes of ncRNAs that are encoded within the human genome is currently unknown but is believed to be numerous.

Functionally, ncRNAs are divided into two main categories: housekeeping ncRNAs, which are involved in generic cellular functions, and regulatory ncRNAs, which primarily regulate gene expression in multiple levels. Hence, their regulatory role in cellular physiology, including normal hematopoiesis, is important, as is their participation in initiation and progression of neoplasia. Indeed, several studies have demonstrated the role of ncRNAs in solid and hematological malignancies, either from a pathophysiologic point of view or as prognostic biomarkers [16,17].

**Figure 1:**
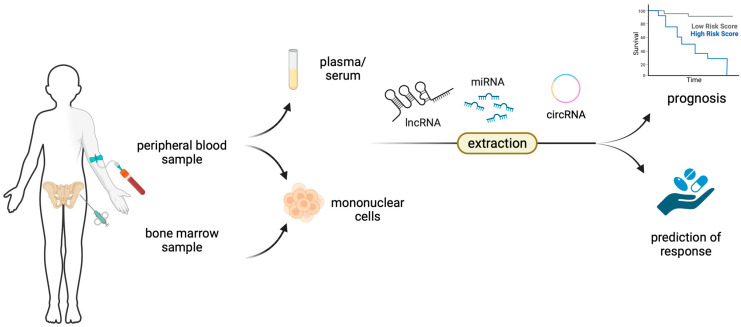
Non-coding RNAs, such as microRNAs (miRNAs), long non-coding RNAs (lncRNAs) and circular RNAs (circRNAs) can be extracted directly from plasma or serum or from mononuclear cells derived from either the peripheral blood or the bone marrow and can serve as prognostic or predictive biomarkers.

In this review, we present a comprehensive summary of findings regarding the emerging role of various ncRNAs in MDS biology, patients’ prognosis and response to therapy. The concept of this manuscript is depicted in Figure 1.

### 1.1. miRNAs in Hematopoiesis and MDS Pathogenesis

MiRNAs belong to a large family of naturally occurring, endogenous, single-stranded ~22-nucleotide-long non-coding RNAs that interact with their target RNA in a sequence-dependent manner, leading to their degradation or translational repression, rendering them significant regulators of posttranscriptional gene expression [18,19]. More specifically, the mature miRNA (derived from pri- and pre-miRNA) binds to the Ago2 protein to form the RNA-induced silencing complex (RISC), which directly suppresses translation of the target mRNA [20]. Each specific miRNA can target multiple mRNAs, while each mRNA may be targeted by several miRNAs. To date, more than 3700 human miRNAs have been identified [21]. MiRNAs are crucial regulators in normal and malignant hematopoiesis [22,23].

HSCs are multipotent, self-renewing progenitors that generate all blood cells [24]. Many genetic and epigenetic regulatory mechanisms are involved in the homeostasis and differentiation of the normal hematopoietic system, including various miRNAs [20,25]. Chen et al. were among the first researchers to identify three miRNAs, namely miR-181, miR-223, and miR-142 that were specifically expressed in hematopoietic cells with a dynamic regulation during the early stages of hematopoiesis. MiRNAs implicated in the self-renewal of HSC in mouse models were miR-33 [26], miR-99 [27], and miR-125a [28]. In addition, at least 33 different miRNAs were found to be expressed in CD34+ HSC playing a role in many different cellular processes and blocking differentiation into mature cells [29]. On the other hand, oncogenic miRNAs (oncomiRs) negatively regulate the expression of tumor suppressor genes, whereas tumor suppressor miRNAs are negative regulators of oncogenes [30,31,32]. The first two oncomiRs that were found to be implicated in cancer were miR-15a and miR-16a in chronic lymphocytic leukemia with deletion 13q14 [33].

Abnormal expression of miRNAs has also been implicated in MDS in various differently prepared samples and using different techniques and statistical methods [34]. For instance, miR-150 plays an important role in the regulation of erythropoiesis and megakaryocytopoiesis and its deregulation has been linked to MDS development [35,36]. The main target of miR-150 is MYB. MYB or c-Myb is a regulatory transcription factor of the hematopoietic system and gastrointestinal tract preserving the balance between cell division, differentiation, and survival [37]. Deregulation of MYB activity has been associated with several hematologic disorders [38]. In a zebrafish model, hyperactivity of MYB led to MDS [38]. In another study, investigators found that MYB was a direct target of miR-150-5p in MDS cells [36]. In these cells, MYB was increased, and its knockdown significantly inhibited cellular proliferation and diminished the proliferation-promoting effect of the inhibitor miR-150-5p [36]. 

Moreover, miR-145 affects megakaryocyte and erythroid differentiation by targeting Fli-1, a megakaryocyte and erythroid regulatory transcription factor [39]. The miR-17-92 is a polycistronic miR cluster, consisting of miR-17, miR-18a, miR-19a, miR-19b, miR-20a, and miR-92a, which is often overexpressed in certain malignancies. This cluster targets the tumor suppressor *PTEN* and the proapoptotic protein Bim by inhibiting their expression [40]. By targeting the proapoptotic protein Bim, miR-17-92 cluster ensures survival of hematopoietic stem and progenitor cells, playing a crucial role in hematopoiesis [41]. Moreover, two other members of miR-17-92 cluster namely miR-17-5p and miR-20a that downregulate E2F1 were found to be underexpressed in high-risk MDS patients constituting favorable prognostic markers associated with increased overall survival (OS) [42]. In the same study, investigators found that let-7a, which downregulates KRAS, was underexpressed in patients with intermediate- or high-risk karyotype [42]. 

MiR-143/145 differentially modulate HSCs and progenitor activity via suppression of canonical tumor growth factor (TGF)-β signaling and loss of expression of these miRNAs can lead to MDS development [43]. The interaction between HSCs, progenitor cells, and bone marrow stromal cells is modulated by CXCL12, a chemokine that is regulated by several different miRNAs [44]. Among them, miR-23a may have a critical role in MDS pathogenesis by regulating the functional properties of the hematopoietic niche [44]. MiR-10a and miR-10b were found to be overexpressed in CD34+ cells, leading to the upregulation of TWIST-1 leading to reduced sensitivity to apoptosis [45]. High levels of miR-21 expression in MDS have been reported to mediate hematopoietic suppression by overactivation of TGF-β signaling [46]. Several tumor suppressor miRNAs, including several let-7 family members, miR-423, and miR-103a, were downregulated in MDS samples with *SF3B1*, *SRSF2*, and *U2AF1* (*U2AF35*) mutations compared to wild type samples, indicating their role in MDS development [47]. In another study, it was shown that upregulation of miR-125a in MDS CD34+ cells modulates NF-kB activation and inhibits erythroid differentiation, rendering miR-125a a potential therapeutic target [48]. This miRNA is supposed to control the size of the stem cells’ pool by modulating their apoptosis [28]. Finally, mutations in the epigenetic modifier *TET2* are involved in the development of myeloid malignancies [49] and are a target of miR-22, a miRNA that is upregulated in MDS [50].

### 1.2. miRNA Deregulation and Cytogenetic Abnormalities in MDS 

Cytogenetic abnormalities are very common in both de novo and secondary MDS [7,51]. The deregulation of several miRNAs has been associated with specific cytogenetic abnormalities. In particular, miR-595 is localized in chromosome 7 and targets RPL27A. It has been found to be downregulated in MDS patients with monosomy 7/isolated loss of 7q (7q-) leading to RPL27A downregulation, p53 activation, apoptosis, and inhibition of proliferation [52]. MiR-205-5p is encoded by chromosome 1, and its upregulation contributes to MDS development via PTEN suppression, causing MDS cells proliferation [53]. Another miRNA that is located in chromosome 1 with its deregulation involved in MDS pathogenesis is miR-194-5p, in MDS patients with trisomy 1 [54].

MDS with isolated del(5q) is characterized by anemia and thrombocytosis [39]. Investigators examined the role of miRNAs that are in this region of chromosome 5 and found that the knockdown of miR-145 and miR-146a resulted in thrombocytosis, mild neutropenia, and megakaryocytic dysplasia [55]. As discussed above, miR-145 affects megakaryocyte and erythroid differentiation by targeting Fli-1, a megakaryocyte and erythroid regulatory transcription factor [39]. Patients with del(5q) MDS were found to have decreased expression of miR-145 and increased expression of Fli-1 [39]. Overexpression of miR-150 was also associated with del(5q) MDS contributing to thrombocytosis [56,57]. In another study, investigators identified 21 different miRNAs that had aberrant expression in del(5q) MDS patients including miR-34a (upregulated), miR-378, and miR-146a (downregulated) [58]. 

The t(2;11)(p21;q23) translocation has been associated with the overexpression of miR-125b, while trisomy 8 was correlated to miR-383 overexpression in MDS patients [59,60]. Kang et al. reported increased expression of miR-661, which is encoded by chromosome 8, in MDS patients via p53 activation [61]. Based on the central role of this protein in tumorigenesis and its potential pharmaceutical targeting, researchers have recently developed molecules capable of stabilizing the oncogenic mutant Y220C of p53 in an effort to overcome its premature degradation and prolong its tumor-suppressive effect, with promising results for clinical use [62]. Another miRNA located on the same chromosome, miR-597, induces apoptosis through downregulation of FOS-like 2 (FOSL2) and was found to be overexpressed in patients with MDS compared to controls, indicating a possible role in MDS pathogenesis [63].

### 1.3. miRNAs as Potential Prognostic Biomarkers in MDS

Many studies have investigated the potential prognostic value of several miRNAs in MDS (Table 1). In one of the first relevant studies, Sokol et al. identified a miRNA signature of ten different miRNAs that was associated with the IPSS risk category and noted the prognostic significance of miR-181 family members in lower-risk MDS patients [64]. Recently, miR-181a-2-3p was shown to be an independent prognostic biomarker in MDS patients in terms of OS [65]. Overexpression of miR-125a was associated with shorter OS and it was found to inhibit erythroid differentiation in leukemia and MDS cell lines [48]. Additionally, miR-22, which targets the TET2 tumor suppressor gene and its overexpression both in plasma and in CD34+ progenitor cells, was associated with high-risk subtypes of MDS, and decreased OS [50,66]. 

Deregulation of many miRNAs is associated with the progression of MDS and transformation to AML, which is a synonym for poor prognosis [67]. Specifically, the upregulation of miR-196b-5p and downregulation of miR-29b have been associated with increased risk of AML transformation [68,69]. Similarly, Kirimura et al. found that the downregulation of miR-29b in MDS bone marrow cells could play a role in the transformation to AML via the upregulation of the antiapoptotic protein myeloid cell leukemia 1 (MCL-1) [69]. Expression levels of miR-422a and miR-617 have also been correlated with disease progression in MDS patients [70]. All members of the miR-320 family (miR-320a, miR-320b, miR-320c, miR-320d, and miR-320e) have been reported to be overexpressed in MDS patients, and in a series of 82 patients, high levels of miR-320c and miR-320d were related to shorter OS, while the upregulation of miR-320d was found to be an independent prognostic factor [71]. 

Furthermore, low levels of miR-194-5p and miR-661 expression have been associated with decreased OS in MDS patients [54,61]. In a cohort of 41 patients, miR-125b-5p, miR-155-5p, and miR-181a-2-3p bone marrow transcript levels were found elevated in higher-risk patients [72] and, likewise, low expression levels of miR-21, miR-126, and miR-146b-5p have been detected in lower-risk compared to higher-risk MDS patients. Among them, elevated levels of miR-126 and miR-155 were associated with shorter OS and leukemia-free survival (LFS), while elevated levels of miR-124a tended to be associated with reduced survival rates [73]. 

Peripheral blood-circulating microRNA profiles have also emerged as useful diagnostic and prognostic biomarkers for MDS patients [74,75]. In particular, the expression levels of miR-27a-3p, miR-150-5p, miR-199a-5p, miR-223-3p, and miR-451a were found reduced in higher-risk MDS patients and the decreased levels of miR-451a and miR-223-3p were independently associated with a lower progression-free survival (PFS) and OS, respectively [75]. Zuo et al. identified and validated a 7-microRNA plasma signature (let-7a, miR-144, miR-16, miR-25, miR-451, miR-651, and miR-655) as an independent predictor of survival in patients with MDS and normal karyotype [74]. Finally, Hrustincova et al. incorporated the expression levels of miR-1237-3p and miR-548av-5p from extracellular vesicles in a prognostic risk score, based on data from 42 patients, as they exhibited the strongest prognostic value in terms of OS [76].

### 1.4. miRNAs as Potential Predictive Biomarkers of Response in MDS

Several studies have attempted to investigate the potential role of miRNAs as predictors of treatment response in patients with MDS (Table 2). Lenalidomide is an immunomodulatory agent that selectively suppresses the del(5q) clone and is used for the treatment of lower-risk MDS with del(5q) [6,77]. Downregulation of miR-145 and miR-146, which are encoded by chromosome 5, plays a crucial role in the development of del(5q) MDS via increased expression of their target genes, TIRAP and TRAF6, respectively, leading to inappropriate activation of innate immune signaling [78]. In a phase II single-arm study in lower-risk MDS patients with anemia, miR-145 and miR-146 were decreased at baseline in patients with del(5q) MDS and significantly upregulated after 3 and 6 months of treatment with lenalidomide [79]. In another study, investigators found that the expression levels of miR-143 and miR-145 were increased during treatment and lenalidomide selectively abrogated progenitor activity in cells depleted of miR-143 and miR-145, rendering them potential predictive biomarkers [80]. Similarly, expression of miRNAs clustering to the 14q32 region and proapoptotic miR-34a and miR-34a* was reduced following lenalidomide administration [81,82]. 

HMAs are nucleoside analogs used for the treatment of higher-risk MDS and the prediction of HMA responsiveness is deemed of critical importance [6]. In a study of 27 patients with higher-risk MDS or AML with myelodysplasia-related changes, the investigators examined the predictive value of specific miRNAs, expressed in bone marrow CD34^+^ cells before and after the administration of azacytidine [83]. Upregulation of miR-17-3p and downregulation of miR-100-5p and miR-133b at baseline was associated with higher overall response rate (ORR) while increased levels of miR-100-5p were associated with shorter OS [83]. Furthermore, deregulation of 30 different miRNAs was observed after the administration of azacytidine in responders. Specifically, miR-10b-5p, miR-15a-5p/b-5p, miR-24-3p, and miR-148b-3p were downregulated in responders after azacytidine treatment while they remained at the same levels in nonresponders, thus rendering them potential predictive biomarkers [83]. Mongiorgi et al. recently showed that miR-192-5p specifically targets and inhibits BCL2 and its overexpression in bone marrow mononuclear cells was correlated to increased OS and leukemia-free survival (LFS) in MDS patients responding to combination of azacytidine and lenalidomide [84]. In a recent study, investigators evaluated the predictive value of miR-22 in MDS patients after HMAs; however, they concluded that it is not an appropriate predictive biomarker [85]. 

Regarding circulating miRNAs in the peripheral blood, miR-21 is a potential predictive biomarker for response to HMA therapy in patients with MDS, since the baseline level of serum miR-21 was found significantly decreased in responders compared to nonresponders [86]. MiR-124 is involved in MDS pathogenesis via targeting the cyclin-dependent kinase 6 (CDK6) gene and was upregulated in response to epigenetic treatments, azacytidine, or the histone deacetylase inhibitor panobinostat in peripheral blood and bone marrow mononuclear cells [87,88]. In another study of 42 MDS patients, investigators identified five circulating miRNAs, namely miR-423-5p, miR-126-3p, miR-151a-3p, miR-125a-5p, and miR-199a-3p, whose combined expression levels in plasma could predict response to azacytidine therapy [76]. Finally, beyond HMAs, in a recent study, investigators found that overexpression of exosomal miR-92a (member of miR17-92 cluster) in plasma promoted cytarabine resistance in MDS/AML by activating the Wnt/β-catenin signaling pathway, rendering miR-92a both a potential predictive biomarker and a therapeutic target for patients with MDS [89].

## 2. Circular RNAs

Circular RNAs (circRNAs) are closed-loop single-stranded RNA molecules that have proved to be important regulators of gene expression at multiple levels although initially considered transcriptional byproducts [90]. CircRNAs function as miRNA sponges or traps that indirectly modulate transcription, interact with intracellular proteins, regulate splicing, and travel in extracellular vehicles called exosomes, enabling intercellular communication [91,92]. In the context of normal hematopoiesis, circRNAs show cell-type specificity and are considered as regulators of blood cell differentiation and maturation [93].

The hypothesis of circRNAs interfering with MDS pathophysiology was supported by the observation that exogenous inhibition of the spliceosome components, commonly affected by MDS mutated genes, can cause an imbalance between circular and linear RNA concentrations within affected cells towards overexpression of the circular molecules [94,95]. Wedge et al. recently reported that specific cancer-associated circRNAs, such as circZNF609 and circCSNK1G3, are upregulated in MDS patients with U2AF1 mutations compared to unmutated controls [96]. Additionally, global circRNA expression has been found to be upregulated in the continuum from normal hematopoiesis to clonal cytopenias of undetermined significance (CCUS) and further to MDS. Even among MDS patients, a higher risk group was correlated with increased global circRNA expression and a “Myeloid Circ Score” was developed based on 14 specific circRNAs with potential prognostic value to stratify patients in terms of risk and disease outcomes [97]. Another research group found 145 circRNAs to be upregulated and 224 downregulated in MDS patients compared to healthy controls. Researchers also suggested that of all these circRNAs, hsa_circRNA_100352, hsa_circRNA_104056, and hsa_circRNA_102817 could be used as MDS prognostic biomarkers, since their increased expression was significantly correlated with poorer OS. Bioinformatics network analysis indicated that these three circRNAs are probably associated with multiple cancer-related molecular pathways, including Wnt/β-catenin and PTEN/Akt/mTOR [98,99]. Additionally, circ-ANAPC7 might be another promising circRNA biomarker, as its expression in MDS patients has recently been shown to be upregulated, along with the increasing risk group, by IPSS-R [100]. Finally, several circRNAs are differentially expressed between responders and nonresponders to azacytidine, although only one circRNA, hsa_circ_0006595, is considered a potential predictor for response to azacytidine treatment [101]. Whether circRNAs will soon be used in clinical practice for diagnostic, prognostic, or predictive purposes remains to be answered, given the need for bone marrow sampling, since the reproducibility of findings in peripheral blood has not been proven yet.

## 3. Long Non-Coding RNAs

Long non-coding RNAs (lncRNAs) are a functionally heterogeneous class of thousands of RNA molecules, each containing more than 200 nucleotides, which are not translated into functional proteins. They are produced through DNA transcription, either from genes or intergenic regions (lincRNAs), and have multiple functions including epigenetic chromatin modifications, regulation of neighboring and distant gene transcription, RNA splicing, response to DNA damage, sponging miRNAs, and participation in signaling pathways [102,103]. In the field of normal hematopoiesis, from murine models to humans, it is known that lncRNAs are expressed in a stage-specific and lineage-specific pattern from hematopoietic stem cells (HSCs) to mature blood cells in a way that they enable self-renewal of HSCs, such as H19 lncRNA, but also determine lineage commitment of progenitor cells, e.g., EGOT lncRNA for eosinophil maturation, in cooperation with transcription factors [104,105,106,107,108,109,110].

After the identification of MEG3 (maternally expressed gene 3) lncRNA hypermethylation in many MDS patients, evidence that linked aberrant expression of lncRNAs with multiple hematological malignancies, including MDS, began to accumulate. The aforementioned lncRNA is considered a tumor suppressor whose downregulation has been associated with poor OS in several solid neoplasms [111,112,113,114,115,116]. While scientific interest in lncRNAs was increasing, researchers identified a positive feedback loop in MDS cells involving lncRNA bc200-miR-150-5P-MYB, which resulted in sustained cell proliferation. On the other hand, the inhibition of this axis seemed to suppress neoplastic growth of bone marrow MDS cells, implying potential therapeutic targeting of BC200 [36]. Additionally, increased expression of the lncRNAs KCNQ10T1 and HOXB-AS3 has been associated with adverse prognosis in MDS, with the latter pertaining to only lower-risk patients [117,118]. Further basic research and computational analysis revealed a vast number of differentially expressed lncRNAs between MDS patients and healthy controls, with functions including cell adhesion, differentiation, and chromatin modifications, mainly through functional interaction with DNA methylation processes [119,120]. Of these lncRNAs, H19 emerged as one of the most promising prognostic biomarkers in MDS patients. Interestingly, a set of 14 lncRNAs were considered as reliable predictive biomarkers to inform about potential patients’ response to azacytidine [101,120,121]. To improve MDS risk stratification by connecting laboratory research with clinical practice, Yao et al. developed a scoring system based on the expression of four lncRNAs with the highest prognostic potential (TC07000551.hg.1, TC08000489.hg.1, TC02004770.hg.1, TC03000701.hg.1). A higher lncRNA score was significantly associated with higher bone marrow blast percentage, higher-risk subtypes by WHO, complex karyotypes, high-risk gene mutations (RUNX1, ASXL1, TP53, SRSF2, and ZRSR2), as well as shorter OS [122]. Consequently, lncRNAs overall appear to be promising prognostic and predictive biomarkers for patients with MDS, probably awaiting their future incorporation in widely accepted prognostic scoring systems to assist in decision-making.

## 4. PIWI-Interacting RNAs

PIWI-interacting RNAs (piRNAs), the third major class of small non-coding RNAs, are single-strand 26–31 nucleotide-long RNA molecules. Their main function, apart from epigenetic modifications, was first believed to be the maintenance of germline DNA integrity through the guidance of PIWI proteins (P-element-induced wimpy testis proteins) towards silencing transposons, which are mobile parasitic genomic elements [123,124]. Further research indicated that aberrant expression of specific piRNAs is associated with the development and progression of several solid and hematological cancers, as these molecules are considered to play a role in continuous proliferative signaling, resistance to apoptosis, tumor invasion, angiogenesis of malignant tissues, and even resistance to antineoplastic treatment [125,126]. On the other hand, though, there has been increasing evidence that aberrant expression of piRNA pathway genes alone might not be adequate for the formation of piRNA–PIWI silencing complexes with biological impact on tumorigenesis [127]. 

Although the importance of piRNAs in other hematological malignancies such as multiple myeloma and classic Hodgkin lymphoma has gathered research interest, data on MDS have been scarce. The first study of piRNAs in bone marrow cells of patients with MDS demonstrated a higher expression (9%) of piRNAs in patients with MDS with refractory anemia (low-risk MDS) compared to patients with MDS with refractory anemia and excess of blasts—2 (high-risk MDS) and healthy controls (2% and 1%, respectively), assuming a DNA-protective role of piRNAs in lower-risk MDS [128,129]. Small non-coding RNA analysis from plasma and extracellular vesicles also showed an upregulation of specific piRNAs (hsa_piR_019914/gb/DQ597347 and hsa_piR_020450/gb/DQ598104) in MDS patients compared to controls. Two other piRNAs, hsa_piR_000805/gb/DQ571003 and hsa_piR_019420/gb/DQ596670, were differentially expressed between patients with low- and increased blasts—MDS. The latter piRNA was also shown to be correlated with OS with a protective role, but no piRNAs were found to have predictive value about patients’ response to azacytidine [76]. The biologic interpretation of these findings as well as the extent to which they can be incorporated in everyday clinical practice remain to be further elucidated. 

## 5. Ribosomal RNAs

Ribosomal RNAs (rRNAs) are indispensable components of ribosomes, the cell’s protein-producing machinery. Ribosomes in human cells comprise four rRNAs (28S, 5S, 5.8S, and 18S) and approximately 80 proteins that are assembled into a small (40S) and a large (60S) subunit through a multilevel process, which mainly takes place in the nucleolus [130,131,132,133].

The dependence of highly proliferative cells, such as the hematopoietic cells, upon protein synthesis has provided the rationale for extensive research on the role of aberrant ribosomal synthesis in several human diseases including hematopoietic neoplasms. In this context, mutation of Nol9 a ribosomal biogenesis protein required for 28S rRNA processing was found to affect hematopoiesis in animal models by reducing proliferation of hematopoietic stem and progenitor cells [134]. Moreover, *DNAJC21* mutations were associated with bone marrow failure with increased tendency to malignancy, attributed to impaired biosynthesis and cytoplasmic maturation of the 60S ribosomal subunit [135]. Similarly, a whole group of diseases termed “ribosomopathies” arising from congenital or acquired genetic abnormalities that lead to impaired ribosomal construction and function have been associated with bone marrow failure and increased risk of hematological malignancies, such as Shwachman–Diamond syndrome or congenital dyskeratosis [136,137]. Further data supporting the correlation of rRNA deregulation with myeloid neoplasms indicate the potential role of *DDX41*, whose germline mutations predispose to myeloid malignancies, in the processing of pre-ribosomal rRNA to mature rRNA [138]. *U2AF1* somatic mutations, commonly detected in MDS patients, apart from altered splicing, are also believed to cause aberrant ribosomal synthesis, mediated by NPM1, which is considered a ribosomal biogenesis factor [139]. Finally, bone marrow CD34+ cells from patients with MDS show decreased rRNA expression compared to controls, which is probably driven by increased promoters’ methylation of DNA loci coding for these rRNAs (rDNA). Interestingly, this hypermethylation can be reversed by hypomethylating agents such as azacytidine and it is therefore implied that methylation status of rDNA could be used as a predictor of response to treatment with such agents, instead of genome-wide methylation status, although this hypothesis is yet to be proven [140,141]. Researchers have recently focused on the study of short RNA fragments cleaved from rRNA, called rRNA-derived fragments (rRFs), as they are believed to regulate cellular functions and show sequence overlap with miRNAs and piRNAs [142,143]. 

## 6. Small Nuclear and Small Nucleolar RNAs

Small nucleolar (snoRNAs) RNAs are 60–300 nucleotide-long RNA molecules derived from coding and non-coding genes and are in the nucleolus of eukaryotic cells. Their main function is processing of other RNA molecules such as ribosomal RNAs and small nuclear RNAs (snRNAs) via pseudouridylation and 2′-O-methylation. In turn, snRNAs are vital components of the spliceosome, the cell machinery that catalyzes pre-mRNA splicing through intron excision and joining of exons, to form functional mature mRNAs [144]. Additionally, snoRNAs are involved in regulation of alternate splicing and also act like miRNAs to selectively suppress gene expression [145,146].

In HSCs, snoRNAs expression is supposed to be cell-type-specific and play an important role in cell homeostasis, self-renewal, and stress response, while their aberrant expression has been linked to several hematological malignancies, MDS included [147,148,149]. For example, *DDX41* regulates snoRNA processing, ribosomal biogenesis, and protein synthesis in hematopoietic stem and progenitor cells (HSPCs) and its germline mutation is known to confer predisposition to clonal myeloid disorders. More specifically, monoallelic DDX41 mutations, as in germline predisposition, increase the risk for age-dependent hematopoietic defects and confer competitive proliferation advantage to HSPCs. On the other hand, biallelic DDX41 mutations deregulate snoRNA processing, causing intracellular accumulation of inappropriately processed snoRNAs; impair protein synthesis; and finally result in cell cycle arrest. Most of the affected snoRNAs belong to the SNORA family and are typically involved in RNA pseudouridylation [150]. Similarly, snoRNA U33, which is a mediator of cell metabolic stress, has been found to be upregulated in MDS patients. More importantly, this snoRNA was shown to be significantly associated with OS of patients, albeit no relevant biological explanation is provided [76,151].

**Table 1:** ncRNAs with prognostic value in MDS.

Class of ncRNAs	ncRNA	Sample	Prognostic Value	Reference
miRNAs	miR-125a	BM	Decreased survival	Gañán-Gómez 2014 [48]
	miR-22	BM and PB (plasma)	Decreased survival	Ma 2020 [66]
	miR-196b-5p	BM	Increased risk of transformation to leukemia	Wen 2017 [68]
	miR-29b	BM	Increased risk of transformation to leukemia	Kirimura 2016 [69]
	miR-320c, miR-320d	BM	Decreased survival	Wan 2021 [71]
	miR-194-5p	BM	Decreased survival	Choi 2015 [54]
	miR-661	BM	Decreased survival	Kang 2019 [61]
	miR-126, miR-155, miR-124a	BM	Decreased survival	Choi 2019 [73]
	miR-181a-2-3p	BM	Decreased survival	Liang 2022 [65] Kontandreopoulou 2022 [72]
	miR-125b-5p, miR-155-5p	BM	Higher risk MDS	Kontandreopoulou 2022 [72]
	miR-451a, miR-223-3p	PB (plasma)	Decreased progression-free survival, decreased survival	Dostalova-Merkerova 2017 [75]
	let-7a, miR-144, miR-16, miR-25, miR-451, miR-651, and miR-655	PB (plasma)	Association of clusters with overall survival	Zuo 2015 [74]
	miR-1237-3p, miR-548av-5p	PB (extracellular vesicles)	Decreased survival	Hrustincova 2020
circRNAs	hsa_circRNA_100352 hsa_circRNA_104056 hsa_circRNA_102817	BM and PB (MNCs)		Wu 2020 [99]
lncRNAs	KCNQ10T1	PB (serum)		Zhang 2020 [117]
	HOXB-AS3	BM		Huang 2019 [118]
	H19, WT1-AS, LEF1-AS, TCL6	BM		Szikszai 2020 [121]
	TC07000551.hg.1 TC08000489.hg.1 TC02004770.hg.1 TC03000701.hg.1	BM		Yao 2017 [122]
piRNAs	hsa_piR_019420	PB (EVs)		Hrustincova 2020 [76]
snoRNAs	U33	PB (EVs)		Hrustincova 2020 [76]
tDRs	tDR-Asp family	FFPE preparations		Guo 2017 [152]

BM: bone marrow; PB: peripheral blood; MNCs: mononuclear cells; EVs: extracellular vesicles; FFPE: formalin-fixed paraffin-embedded.

## 7. Transfer RNAs and Their Derived Fragments

Transfer RNAs (tRNAs), with their unique stem–loop pattern formed by internal base pairing, are essentially the carriers of amino acids to the growing polypeptide chain at the ribosome during translation but are also believed to have additional functions such as modulation of gene expression and control of cell death. Cleavage of pre- or mature tRNAs produces the tRNA-derived fragments (tRFs) or tRNA-derived small RNAs (tsRNAs) or tRNA-derived RNAs (tDRs), which are involved in multiple biological processes including translational regulation with gene silencing, intercellular communication, cellular stress response, and immune cell activation, rather than being useless byproducts of tRNA degradation [153,154,155,156]. 

Specific tRNAs (chr2.tRNA27-GlyCCC, chr.18Trna4-LysCTT) as well as overall tRNA to rRNA ratio have been found upregulated in marrow cells from MDS patients compared to controls, and it was assumed that this increase might contribute to decreased programmed cell death and increased leukemic transformation, since tRNAs are known to inhibit cytochrome c activated apoptosis [76,107,128,157]. On the other hand, the SF3B1^K700E^ mutation commonly seen in MDS seems to reduce translational machinery components, primarily tRNA synthetases [158]. Another somatic mutation in the mitochondrial tRNA repertoire, MtRNA^Leu(UUR)^, in bone marrow cells is suspected to contribute to ineffective hematopoiesis [159].

When it comes to tRFs, some of them show enhanced expression while others are downregulated in MDS cells. Interestingly, the combined expression of 4 tRFs (chr6.tRNA157.ValCAC, chr11.tRNA17.ValTAC, chrM.tRNA12.TS1, and chrX.tRNA4.ValTAC) in treatment-naïve patients was found to have predictive value regarding the likelihood of response to treatment, and this is also the case with one mitochondrial tRNA (MT-TSI), while it is suggested that tDR-Asp family members could be used as predictors for progression to AML [152,160].

Even posttranscriptional modifications of these non-coding RNAs are suspected to interfere with MDS pathophysiology. Pseudouridylation by PUS enzymes, for instance, of mini tRFs containing 5-terminal oligoguanine, was found to regulate the renewal of human embryonic stem cells and also promote the differentiation of impaired HSPCs in MDS, indicating a potential therapeutic approach [161,162,163].

## 8. Short Interfering RNAs

Short or small interfering RNAs (siRNAs) are 21–25 nucleotide-long RNA molecules with a crucial role in gene silencing, primarily through mRNA degradation and by promoting heterochromatin formation. These interfering RNAs are produced via the procession of long double-stranded RNAs or short hairpin RNAs by the DICER endoribonuclease. The produced double-stranded siRNA is then packed with proteins to form the RISC. One strand of the RNA is discarded, and the remaining strand guides the RISC towards the targeted mRNA, which is recognized with perfect complementarity with the siRNA and is finally cleaved by Ago2 protein of the RISC [164,165,166]. 

The well-established way of action of RNA interference has not only made it possible for researchers to better understand its implications in cancer pathogenesis but also provided the possibility to utilize siRNAs towards gene expression knockdown with research and therapeutic purposes. For instance, siRNAs have been used in basic research as tools to knockdown expression of genes that are commonly mutated in MDS patients, such as ZRSR2 and antiapoptotic “survivin”, so as to better investigate their role in MDS pathophysiology [167,168]. Additionally, Mackin et al. showed that compared with azacytidine, which is a hypomethylating pharmacologic agent, siRNAs targeting *DNMT* expression (DNA methyltransferase) proved more efficient at overall demethylation within the genomic transcription units [169]. Another clue to the potential therapeutic role of siRNAs came when the siRNA-mediated inhibition of p38*a* MAP kinase, a mediator of apoptosis that is constitutively activated in low-risk MDS bone marrow cells, led to in vitro improvement of hematopoiesis from MDS myeloid and erythroid progenitors [170]. It is therefore implied that siRNAs could provide a means of therapeutically targeting multiple genes that are aberrantly expressed in MDS patients, although no such agents have been tested in MDS patients to date.

**Table 2:** ncRNAs with predictive value of treatment response in MDS.

Class of ncRNAs	ncRNA/Gene	Sample	Reference
miRNAs	miR-143, miR-145	BM	Venner 2013 [80]
	miR-145, miR-146	BM	Oliva 2013 [79]
	miR-34a, and miR-34a*	PB (MNCs)	Merkerova 2015 [82]
	miR-17-3p, miR-100-5p, miR-133b miR-10b-5p, miR-15a-5p/b-5p, miR-24-3p, miR-148b-3p	BM	Krejcik 2018 [83]
	miR-124	BM	Wang 2017 [87]
	miR-21	PB (serum)	Kim, 2014 [86]
	miR-423-5p, miR-126-3p, miR-151a-3p, miR-125a-5p, miR-199a-3p	PB (plasma)	Hrustincova 2020 [76]
	miR-192-5p	BM and PB (MNCs)	Mongiorgi 2023 [84]
	miR-92a	PB (plasma)	Li 2022 [89]
circRNAs	hsa_circ_0006595	BM	Merkerova 2022 [101]
lncRNAs	AC010127.5, CTC-482H14.5, RP11-557C18.3, RP4-580N22.1, RP11-419K12.2, MIR4512, MIR3164, RF00019, RPS6P16, RP11-478C6.2, RP11-177A2.5, RP4-740C4.7, AC097382.5, RP11-736I24.4	BM	Merkerova 2022 [101]
tRNA/tDRs	chr6.tRNA157.ValCAC chr11.tRNA17.ValTAC chrM.tRNA12.TS1 chrX.tRNA4.ValTAC MT-TS1 chr1.tRNA35.GlyGCC chr21.tRNA2.GlyGCC chr19.tRNA9.PseudoTTT	BM	Guo 2015 [160]

BM: bone marrow; PB: peripheral blood; MNCs: mononuclear cells.

## 9. Conclusions

Myelodysplastic neoplasms are very heterogenous in terms of genetic and epigenetic background, clinical presentation, and prognosis. Treatment decisions are mainly based on the risk stratification of the patients with the use of validated prognostic scoring systems such as IPSS-R and most recently IPSS-M. Yet, more biomarkers are needed not only to assess prognosis but also to predict response to therapy. Non-coding RNAs, mostly miRNAs, have been found to be implicated in normal and malignant hematopoiesis including MDS. Their role as prognostic and predictive biomarkers is beginning to emerge and deserves to be further evaluated in large number of patients. Moreover, it is important that experiments are performed in well-preserved and well-defined samples so that reliable data are generated and safe conclusions drawn.

## Data Availability

No new data were created.
